# Tree-related microhabitats harbor distinct micro-invertebrate communities and support complex food webs

**DOI:** 10.1007/s00442-025-05774-5

**Published:** 2025-08-21

**Authors:** Nabil Majdi, Walter Traunspurger, Joseph Garrigue, Laurent Larrieu

**Affiliations:** 1 Massane Forest Reserve, Banyuls-sur-Mer, France; 2 Toulouse University, LRSV, UMR 5546 UPS/CNRS/INPT, Auzeville-Tolosane, France; 3 Sciences and Arts of Southern Switzerland, Institute of Microbiology, University of Applied, Mendrisio, Switzerland; 4 Animal Ecology, Bielefeld University, Bielefeld, Germany; 5 INRAE, UMR DYNAFOR, Toulouse University, Auzeville-Tolosane, France; 6 CNPF-CRPF Occitanie, Tarbes, France

**Keywords:** Biogeography, Trophic ecology, Invertebrates, Biodiversity, Conservation

## Abstract

Microscopic soil invertebrates are known to play an important role in forest ecosystems through their interactions with the rhizosphere and belowground food webs. However, little is known about the abundance, diversity, distribution, and ecological roles of micro-invertebrates above the forest floor, particularly within tree-related microhabitats (TreMs). In this study, we sampled 18 distinct types of TreMs in the UNESCO World Heritage old-growth beech forest of La Massane, located in the southeastern Pyrenees. We extracted and counted various groups of micro-invertebrates (nematodes, tardigrades, rotifers, and mites) associated with these TreMs, and identified nematodes to the species level. Additionally, we measured the stable isotopic signatures of carbon and nitrogen in various resources and invertebrate groups to reveal the structure of TreM-associated food webs in unprecedented detail. TreMs emerged as hotspots of both abundance (averaging 195 individuals per gram of dry TreM substrate) and diversity (98 nematode morphospecies across 20 families). We found significant differences among TreM types in terms of community composition and food web structure, revealing distinct species assemblages and trophic pathways. These differences could be linked to factors such as the stage of wood decomposition, occupation by larger animals (e.g., insects, birds), and the position of TreMs within trees. Our findings suggest that micro-invertebrates serve as valuable ecological indicators, adding a new layer of biodiversity and functional understanding to TreM typology. This, in turn, supports more comprehensive strategies for nature conservation and forest management.

**Supplementary Information:**

The online version contains supplementary material available at 10.1007/s00442-025-05774-5.

## Introduction

Tree-related microhabitats (TreMs) are distinct, well-defined structures such as cavities, crown microsoils and epiphytic formations that occur on living or standing dead trees and host species or communities for at least part of their life cycle (Larrieu et al. 2018). TreMs are increasingly recognized as critical components of forest biodiversity (e.g., Winter and Möller 2008; Paillet et al. 2018; Basile et al. 2020). As such, several authors have proposed using TreMs as indirect indicators of species richness (e.g., Paillet et al. 2018; Larrieu et al. 2019; Asbeck et al. 2021) and as practical tools to promote biodiversity-friendly forest management (Bütler et al. 2013).

TreMs have been classified into seven main forms, 15 groups and 47 distinct types based on their morphology and associated species assemblages (Larrieu et al. 2018). A wide range of taxa have been linked to different TreM-types, including vascular plants, fungi, invertebrates (particularly insects), gastropods, arachnids, and vertebrates such as bats, birds, rodents, and carnivores (Larrieu et al. 2018; Bütler et al. 2024). Over the past few decades, TreMs have received growing attention in ecological research (reviewed in Martin et al. 2022). Some TreM-types and their typical species associations are now well documented: e.g., woodpecker cavities (Martin and Eadie 1999; Cockle et al. 2011), rot-holes (Ranius et al. 2002) and water-filled tree-holes (dendrotelms; Kitching 1971; Petermann and Gossner 2022). However, knowledge gaps remain concerning TreM-associated species both at the scale of individual TreMs and at broader stand levels..

At the TreM scale, micro-invertebrates remain particularly understudied. While some research has highlighted diverse communities of nematodes, rotifers, tardigrades, and mites within specific TreM-types (e.g., mosses: Merrifield and Royce 2002, Nelson et al. 2019; bark: Majer et al. 2003; dendrotelms: Ptatscheck and Traunspurger 2014; lichens: Bokhorst et al. 2015). However, no study to date has examined the distribution and ecological roles of micro-invertebrates across the full spectrum of TreM-types associated with a single tree species. TreMs are also inherently transient and patchy resources (Finn 2001), with lifespans ranging from a few days (e.g., ephemeral fungal fruiting bodies), to several decades (e.g., large rot-holes) (Ranius et al. 2009). TreMs are shaped by various dynamic processes such as fungal colonization and maturation, wood decomposition rate, or excavation of cavities by animals, and all those changes influence the composition of resident communities (Stokland et al. 2012). Due to their rapid population turnover and their sensitivity to resource availability (e.g., Maraun et al. 2001; Majdi and Traunspurger 2017), micro-invertebrates may serve as particularly relevant indicators of TreMs functioning and successional stage.

At the stand-scale, relationships between TreMs and their associated taxa are complex. Overlaps between resources provided by external inputs, by deadwood or by saproxylic organisms (Larrieu 2014), may contribute to the limited and often weak correlations observed in broader-scale studies (Larrieu and Bouget 2017). Asbeck et al. (2021) emphasized the need for a multi-taxon approach to better assess the relationships between forest-dwelling and TreM-dwelling species and thus improve the scientific basis for TreM-based conservation strategies.

In forest ecosystems, soil micro-invertebrates (especially nematodes) are known to play important functional roles in nutrient cycling and food web dynamics (e.g., Yeates 2007; Wagg et al. 2014; Trap et al. 2016). Their taxonomic and functional diversity has emerged as a useful indicator of forest maturity (e.g., Zhang et al. 2015). A recent molecular survey by Ahmed et al. (2024) stressed the importance of sampling a broad range of soil microhabitats to accurately assess soil diversity in a forest reserve. Yet, the diversity and ecological roles of nematodes inhabiting aboveground micro-habitats, such as TreMs, remain largely unexplored.

European beech (*Fagus sylvatica* L.) is a dominant species in many temperate broadleaf forests across Europe and supports a rich diversity of associated fungi and animals (e.g., Mueller et al. 2013). Compared to other species, beech trees tend to develop a wide diversity of TreMs early in their lifespan (Courbaud et al. 2022).

In this study, we investigated the diversity, distribution, ecological traits, and stable isotopic signatures (i.e., trophic positioning) of micro-invertebrates inhabiting most of TreM-types found on European beech. Data were collected in the Massane Forest Reserve, a UNESCO World Heritage site, featuring an old-growth Mediterranean beech forest in southern France that has been extensively studied for over 70 years for its remarkable flora and fauna (e.g., Travé et al. 1954; Auger et al. 2023; Majdi et al. 2024). The aims of this study were to (1) Quantify the abundance and diversity of TreM-dwelling micro-invertebrates. (2) Assess how species composition and functional traits vary in response to different TreM-types, thereby enriching the existing TreM-typology (Larrieu et al. 2018). (3) Examine how food web structure and trophic pathways differ across TreMs, depending on the diversity and availability of resources.

## Material and methods

### Study site

**Fig. 1:**
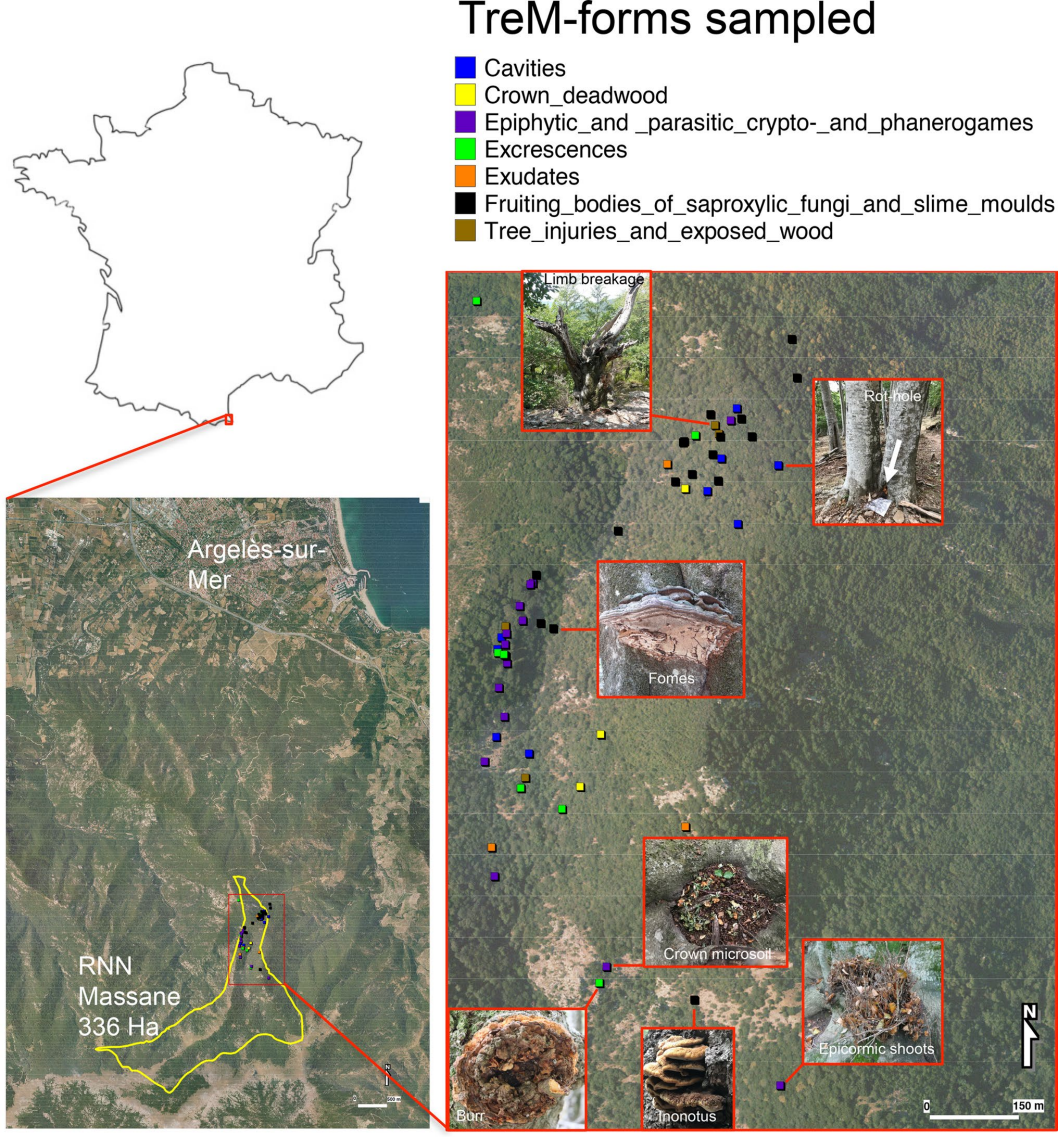
Map of the Massane Forest reserve in southern France showing the locations of the broad categories of tree-related microhabitats (TreM-forms) sampled in this study. Insets on the map show examples of TreM-types and their localization

The Massane Forest Reserve is a mountainous old-growth beech forest located at a biogeographical intersection of Pyrenean and Mediterranean regions (Fig. 1). Spanning 336 hectares, this forest has experienced minimal human disturbance for over 150 years. It has held French National Nature Reserve since 1973 and was designated UNESCO World Heritage site in 2021. The Massane Forest Reserve is characterized by unaltered, natural dynamics of tree growth, death and decomposition since several centuries, offering a unique setting for studying'pristine'forest biodiversity and ecosystem processes.

### Sampling

This study focused on tree-related microhabitats (TreMs) commonly associated with European beech (*Fagus sylvatica* L.) within the core protected area of the Massane Forest Reserve. Based on prior field surveys, we selected the most accessible and representative TreM-types. In 2021, we collected a total of 54 samples from 18 different TreM-types, with three replicates per type, each taken from a different individual tree. Sampling was conducted across a 20-hectare area at elevations ranging from 600 to 700 m (Fig. 1; Table S1 in Supplementary Material).

The 18 TreM-types were categorized into six broader TreM-forms following the classification of Larrieu et al. (2018). Sampling was carried out across three seasons (spring, summer, and Autumn 2021) depending on the accessibility and seasonal availability of certain TreMs (see Table S1 for details).

For each TreM sample, we collected approximately 100 g of soft substrate using a spoon or small shovel. In the case of hard-substrates, tools such as knives, hand axes, or small saws were used to carefully collect part, or all, of the TreM from the tree (Table S1). Samples were immediately sealed in airtight bags and transported to the laboratory within 24 h.

### Extraction of micro-invertebrates

Micro-invertebrates were extracted from TreM samples using a modified Baermann funnel technique, based on Travé et al. (1954) (see Supplementary Material for detailed protocol). Following extraction, the organisms were resuspended in 20 mL milliQ water. Half the volume of the initial suspension (10 mL) was preserved in a final solution of 4% buffered formaldehyde, with a few drops of Rose Bengal. This first half-suspension was used to quantify the abundances of micro-invertebrates at the phylum-level, and then mount nematode specimen on slides to identify them at species-level, and group them into different morphofunctional categories.

The other half of the suspension (10 mL) was not fixed with any preservatives, but rather directly kept frozen at –20 °C for stable isotopic analyses (SIA). After extraction, remaining TreM samples were inspected for the presence of macro-invertebrates, which were picked out, sorted at family or genus-level, starved in individual glass vials for 24 h and individually stored at –20 °C for SIA. Resulting defaunated TreM-samples were dried in a oven at 40 °C during 7 days, weighed, grinded to powder and stored at –20 °C as a source of coarse-particulate organic matter (CPOM) for stable isotopic analyses.

### Micro-invertebrate abundances, morpho-taxonomy and trait-based indices

Formaldehyde-fixed micro-invertebrates were counted under a stereomicroscope (40–80x) and first determined at a coarse taxonomic-level (mostly nematodes, rotifers and tardigrades). Abundances were expressed relative to the dry weight of each TreM sample. A minimum of 200 individuals was counted per sample/sub-sample. While counting, the first 100 nematode individuals encountered were picked out, transferred to glycerol, and mounted on slides after Seinhorst (1959). Mounted nematode specimen (total number of nematodes identified: 4985, i.e., mean of 92 nematodes sample^–1^) were identified using a compound microscope to species level when possible (1250-fold magnification; Leitz, Dialux), following relevant literature, and then further classified into different feeding-types and functional-trait categories based on their size, life-history traits and the morphology of their buccal cavity (Traunspurger 1997; Hodda 2022; Preez et al. 2022).

### Stable isotopic analysis

When present in sufficient numbers (i.e., in 11 out of the 18 TreM types; see Table S1), nematodes and other micro-invertebrates (including tardigrades, rotifers, mites, and diptera larvae) were extracted from thawed samples under a stereomicroscope (40–80x) and grouped according to practical taxonomic or trait-based categories. Individuals or groups of invertebrates were carefully picked, rinsed in successive milliQ water baths to remove adhering particles, and placed in pre-weighted tin capsules (see Supplementary Material for detailed procedures). Basal TreM CPOM and previously starved macro-invertebrates were similarly encapsulated. All tin capsules were dried at 40 °C for 3 days, sealed and weighed to the nearest 10 μg prior to stable isotope analysis (^13^C/^12^C and ^15^N/^14^N) using an elemental analyzer coupled to an isotope ratio mass spectrometer optimized to low carbon and nitrogen content (details in Supplementary Material).

### Data analysis

All statistical analyses were conducted using R version 4.0.3 (R Development Core Team 2020). Univariate community descriptors (including abundance, diversity and functional indices) were tested for homoscedasticity and normality using Levene’s and Shapiro–Wilk tests. Data not meeting these assumptions were log_10_-transformed. One-way ANOVAs were performed to assess differences across TreM-forms (six groups) and TreM-types (18 groups), followed by Tukey’s HSD post hoc tests for pairwise group comparisons.

To account for varying numbers of individuals identified per sample, species richness was estimated using the Chao1 estimate (Chao 1987). The observed mean species richness per sample (9.22) closely matched the Chao1 estimate (9.27), indicating that the sampled individuals provided a robust representation of TreM communities. Additional diversity metrics, including evenness and Pielou’s equitability were also computed and compared.

Functional structure of nematode communities was assessed using several indices: Bonger’s Maturity Index (MI: Bongers 1999), Heip's Index of Trophic Diversity (ITD: Heip et al. 1985), and Ferris’ Nematode Channel Ratio (NCR), calculated as the ratio of bacterivores to bacterivores plus fungivores (Bongers and Bongers 1998).

Community structure differences among samples were tested using Permutational Multivariate Analysis of Variance (PERMANOVA), with Bray–Curtis similarity as the response metric (9,999 permutations), implemented via the *adonis* function in the ‘vegan’ package (Oksanen et al. 2020). The *rankindex* function was used to evaluate dissimilarity metrics, with Bray–Curtis identified as the most appropriate in all cases. P-values were adjusted for false discovery rate (α = 0.05) using the Benjamini–Hochberg method. Homogeneity of multivariate dispersion was verified using the PERMDISP2 procedure (Anderson 2006). Post hoc pairwise comparisons among sample groups were conducted using the *pairwise.adonis2* function from ‘pairwiseAdonis’ package (Arbizu 2017). Sample similarities were visualized via non-metric multidimensional scaling (NMDS) based on Bray–Curtis dissimilarity.

Species-level responses to TreM-forms and TreM-types were examined through multi-level pattern analysis using the *multipatt* function in the ‘indicspecies’ package, following Cáceres & Legendre (2009), to identify taxa contributing significantly to observed community differences.

Isotopic niches of functional groups and entire communities within the three main TreM-forms (cavities, fungi, and epiphytes) were analyzed using the Bayesian framework of the ‘SIBER’ package (Jackson et al. 2019). The assumption of multivariate normality of isotope data was tested using the Shapiro–Wilk test. Since some groups did not meet this assumption, isotopic niche analyses were restricted to three broad functional categories: coarse particulate organic matter (CPOM, as a basal resource), micro-invertebrates (including nematodes, rotifers, tardigrades, mites, springtails), and macro-invertebrates (including insect larvae/adults and diplopodes). ‘SIBER’ was used to calculate Bayesian versions of Layman's metrics to describe and compare the isotopic space occupied by different TreM communities (details in Supplementay Material).

## Results

### Micro-invertebrate abundance and composition

**Fig. 2:**
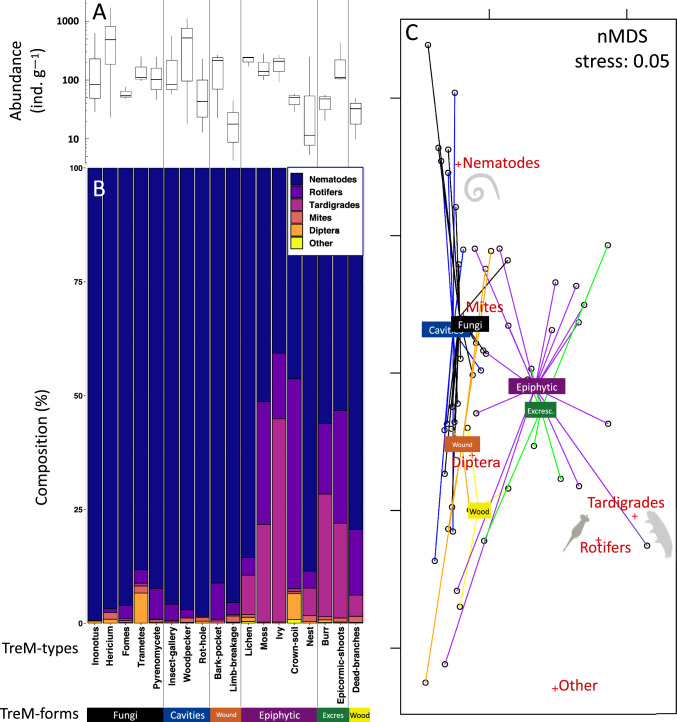
**A** Total abundance of micro-invertebrates across different types and forms of tree-related microhabitats (TreMs) in the Massane forest reserve (*N* = 3 per TreM-type; boxplots show medians, first, and third quartiles). **B** Relative community composition (%) of micro-invertebrates across TreMs. **C** Non-metric multidimensional scaling (nMDS) based on Bray–Curtis dissimilarity showing differences in community composition across TreM-forms. Spider plots link sample scores to their group centroids; species scores are also displayed. Silhouettes indicate representative micro-invertebrate groups (nematodes, rotifers and tardigrades)

Micro-invertebrates were found in all TreMs, with an average abundance of 195 individuals per gram of dry substrate (Fig. 2A), ranging from 4 to 1698 ind. g^–1^. Despite this variability, total abundance did not differ significantly across TreM-forms (ANOVA: F_5,49_ = 0.9, *P* = 0.44) or TreM-types (ANOVA: F_17,37_ = 1.4, *P* = 0.19).

Communities were dominated by nematodes (80%), followed by rotifers (10%) and tardigrades (7.5%), with mites and dipterans contributing < 1% (Fig. 2B). Community composition differed significantly between TreM-forms (Fig. 2C, PERMDISP2, *P* = 0.63; PERMANOVA: F_5,48_ = 1.8, R^2^ = 0.15, *P* = 0.027), with ‘fungi’ and ‘cavities’ dominated by nematodes (96%), and ‘epiphytes’, ‘excrescences’, and ‘crown deadwood’ hosting more rotifers and tardigrades (Fig. 2C).

### Nematode diversity and functional traits

**Fig. 3:**
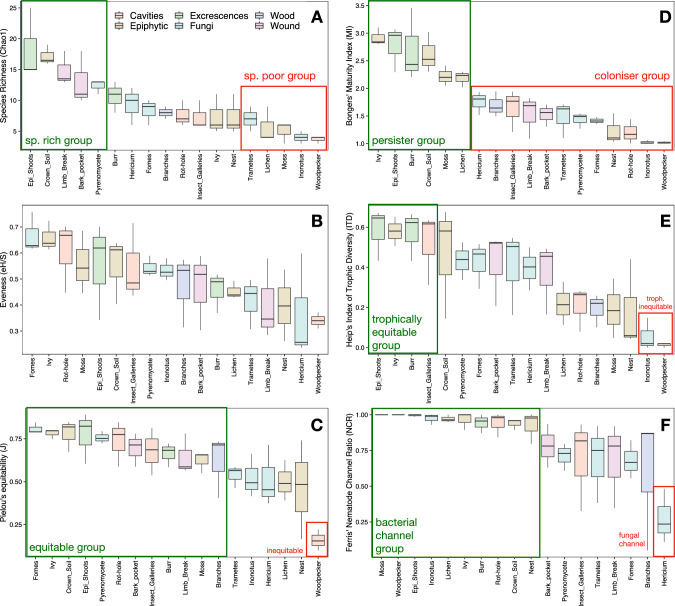
Comparison of nematode diversity and functional indices across TreM-types. Boxplots (*N* = 3) show medians, first, and third quartiles; box colors correspond to TreM-form categories. Green and red boxes indicate significantly higher or lower group values, respectively (ANOVA, *P* < 0.05 followed by Tukey's HSD post-hoc). TreM-types are ordered by decreasing mean value for each variable. **A** Chao-1 estimated species richness. **B** Evenness. **C** Equitability. **D** Maturity Index. **E** Trophic Diversity. **F** Nematode Channel Ratio

A total of 4985 nematodes were identified, spanning 98 morphospecies and 20 families, with four families (Rhabditidae, Plectidae, Aphelenchoididae and Tylenchidae) accounting for 50% of species (Table S2). Species richness varied significantly across TreM-types (Fig. 3A; ANOVA, F_17,36_ = 7.5, *P* < 0.001), with epicormic shoots, crown microsoils, limb breakage, bark pockets and pyrenomycetes hosting richer communities than types like polypores, lichens, mosses and woodpecker breeding cavities. The latter was dominated (> 93%) by a single small bacterivorous Rhabditid species (*Mesorhabditis spiculigera*), resulting in highly uneven assemblages (Fig. 3C, ANOVA, F_17,36_ = 4.8, *P* < 0.001).

Functionally, communities were dominated by opportunistic bacterivores (67%), followed by fungivores (16%), phytoparasites (6%), predators (5.5%), and omnivores (5.3%). Significant differences were observed in Maturity Index (MI) and Index of Trophic Diversity (ITD) across TreM-types (ANOVA, *P* < 0.001; Fig. 3D–E). Woodpecker breeding cavities and *Inonotus* types showed the lowest MI and trophic diversity, while ivy spike roots, epicormic shoots, burr, crown microsoils, mosses and lichens hosted more mature, functionally diverse communities. Nematode Channel Ratio revealed dominance of the bacterial-feeding channel across most TreMs, except in *Hericium*, where fungal-feeding prevailed (ANOVA, F_17,36_ = 3.38, *P* = 0.001; Fig. 3F).

### Nematode community structure and indicator species

**Fig. 4:**
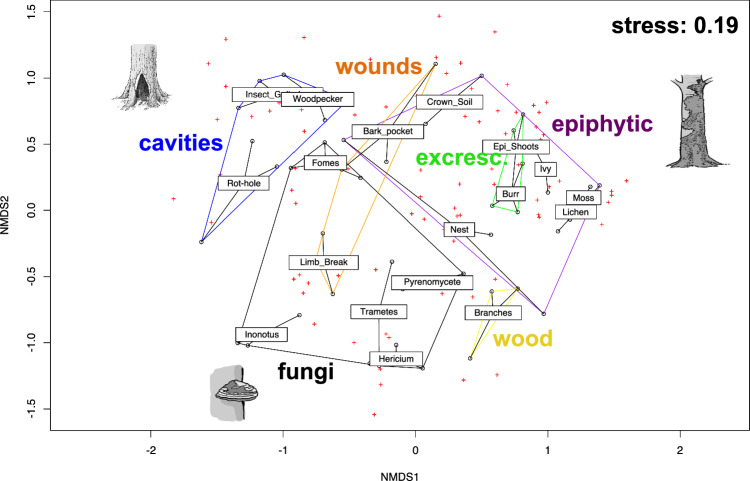
Nonmetric multi-dimensional scaling (NMDS) of nematode community (Bray–Curtis dissimilarity) across 6 TreM-forms and 18 TreM-types found on European beech trees in the Massane Forest Reserve. Lines connect samples to their TreM-type centroids. Colored hulls indicate TreM-form grouping. Crosses represent nematode species scores. TreM illustrations adapted from Kraus et al. (2016)

Nematode community structure varied significantly among both TreM-forms (PERMDISP2, *P* = 0.07; PERMANOVA: F_5,48_ = 5.44, R^2^ = 0.36, *P* = 0.001) and TreM-types (PERMDISP2, *P* = 0.14; PERMANOVA: F_17,36_ = 8.02, R^2^ = 0.79, *P* < 0.001) (Fig. 4). Distinct assemblages were identified for most TreM-types, except for overlap between mosses and lichens and between burrs and epicormic shoots. Bird nest communities were more variable and lacked clear differentiation.

Multi-level pattern analysis identified 43 species as indicators of specific TreM-types (Table S2). Examples include *Bursilla monhystera* in bark pockets, *Aphelenchoides fragariae* in crown deadwood, and *Diplogasteritus nudicapitatus* for rot-holes. Some TreMs hosted characteristic multi-species assemblages such as Dorylaimid and Plectid nematodes in crown microsoils, mosses and lichens.

### Stable isotope signatures of sources and consumers

Carbon and nitrogen isotope signatures revealed distinct food web structures among TreM-types (see Fig. S1, and details in Supplementary Material). In cavities, food webs differed between rot-holes and woodpecker cavities, the latter featuring highly ^15^N-enriched signatures of sources and consumers. The isotopic signatures of mosses and lichens diverged sharply, but predatory nematodes, tardigrades, and mites similarly dominated both food webs, as well as in other epiphytic TreM-types such as ivy spike roots and crown microsoils. Among fungal TreMs, we observed relatively less complex webs based on fungal tissues as basal resource fueling directly the fungivorous nematodes and/or insect larvae. Bacterivorous nematodes showed unexpectedly enriched ^15^N signatures suggesting they may be able to exploit the bacteria thriving on insect carcasses, feces or'frass'(Fig. S1).

### Isotopic niches and food web metrics

**Fig. 5:**
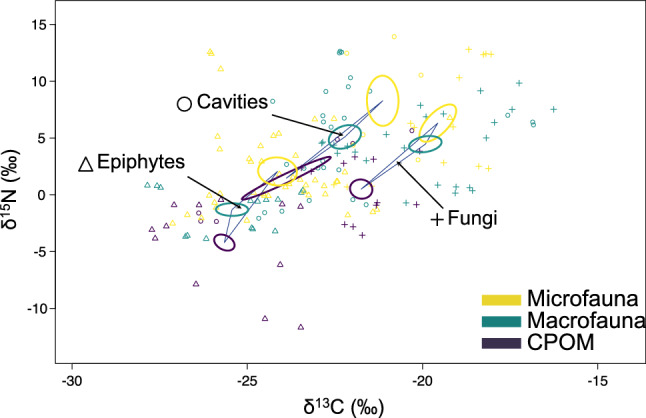
Stable isotope signatures (δ^13^C and δ^15^N) of basal resources (CPOM), microfauna (nematodes, rotifers, mites, springtails), and macrofauna (insect larvae/adults, diplopods) across three main TreM-forms (‘epiphytes’, ‘cavities’, and ‘fungi’) in an old-growth Mediterranean beech forest. Ellipses represent 50% confidence intervals of bivariate means for each group. Triangular hulls connect group centroids within each TreM-form, delineating the isotopic niche space associated with that of a particular TreM-form

**Fig. 6:**
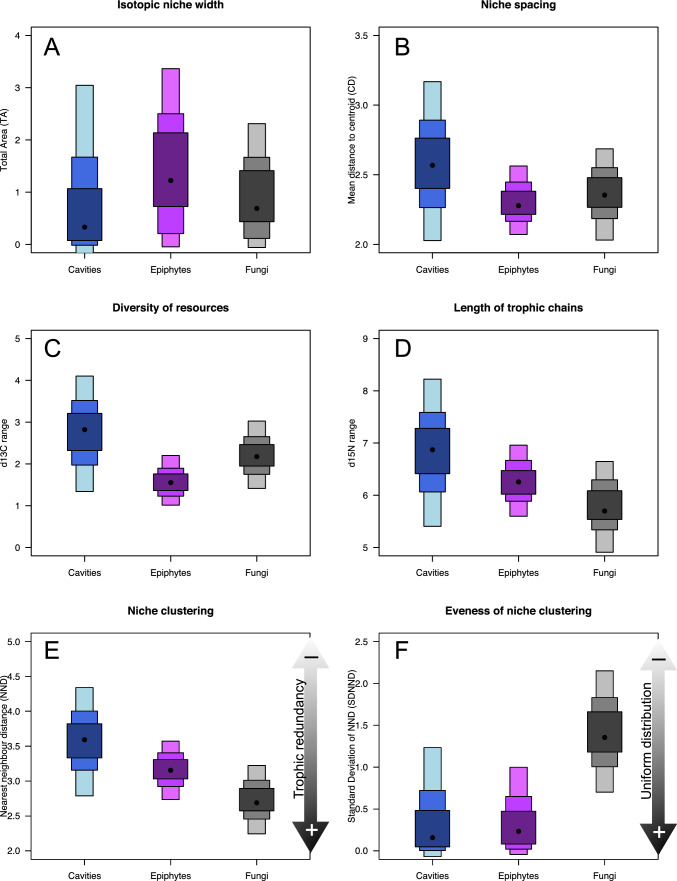
Layman et al. (2007) metrics describing the isotopic niche space occupied by communities in three TreM-forms (‘epiphytes’, ‘cavities’ and ‘fungi’) using Bayesian estimates (Layman.B). Black dots represent modal values; shaded areas show 50, 75 and 95% credible intervals. Differences in Layman.B modes reflect the posterior probability of differences between groups. Panels: **A** Total isotopic niche area (TA: convex hull area in δ^13^C–δ^15^N space). **B** Niche spacing (CD: mean Euclidean distance to community centroid). **C** Resource diversity (CR: maximum δ^13^C range). **D** Trophic chain length (NR: maximum δ^15^N range). **E** Niche clustering (NND: mean nearest neighbor distance, with small NND values indicating high trophic redundancy). **F**. Evenness of niche clustering (SDNND: standard deviation of NND, with small SDNND values indicating more uniform distribution of niche clusters)

The isotopic niches of ‘cavities’, ‘epiphytes’, and ‘fungi’ were non-overlapping (Fig. 5). Micro-invertebrates consistently occupied top trophic positions. ‘Epiphytes’ displayed the most ^13^C-depleted signatures and the largest niche area, though with narrower ^13^C range (Fig. 6A–C).'Cavities' had greater niche dispersion, resource diversity, trophic chain length (Fig. 6B–D), and lower trophic redundancy (Fig. 6E), suggesting more specialized food webs. In contrast, ‘fungi’ had higher trophic redundancy and clustered isotopic niches (Fig. 6F).

## Discussion

### Abundance and diversity of TreM-dwelling micro-invertebrates

This study revealed that tree-related microhabitats (TreMs) support rich and abundant communities of micro-invertebrates, with a special focus on nematodes, underscoring their ecological relevance beyond the soil compartment. In terms of abundance, TreMs housed remarkably high numbers of micro-invertebrates compared to forest soils. We recorded a mean of 14,179 nematodes per 100 g of TreM dry weight, which is three times higher than average nematode abundance in temperate broadleaf forests soils, and 23 times higher than in Mediterranean soils (Van Den Hoogen et al. 2019). As observed in soils, abundance varied substantially across different TreM-types, highlighting the importance of extending investigations across broader spatial and temporal scales. Considering TreM-dwelling micro-invertebrates will surely improve our general understanding of animal abundance and biomass distributions on Earth, however, a critical knowledge gap remains, regarding the quantification of TreM occurrence and distribution across distinct forests. Larrieu et al. (2022) highlighted how TreM distribution is shaped by site-level (e.g., wind, fertility), stand-level (e.g., tree density), and tree-level (e.g., age, health, architecture) factors, which in turn influence the presence and composition of their associated faunal assemblages. We recommend further research including a large range of sites and organisms to assess the broader ecological significance of TreM-dwelling fauna. To efficiently assess TreM-associated assemblages at high throughput, environmental DNA offers a promising approach for measuring diversity (e.g., Ahmed et al. 2024), while automated image acquisition and recognition provide a time-effective means of quantifying abundances and assessing key morphological traits of the community (Saikai et al. 2024). Particularly for TreM surveys, since Baermann-funnel extraction yields relatively clean samples readily usable for image automation.

Tardigrades, rotifers and nematodes were the most frequently encountered groups, consistent with their ability to colonize a wide range of forest microhabitats. Although rotifers and tardigrades were not identified to species-level in this study, other surveys in nearby limno-terrestrial habitats reported 67 rotifer and 25 tardigrade species (Majdi et al. 2024). For nematodes, we identified 98 species, aligning with previous reports from forest soils, litter, mosses, and lichens in temperate forests (e.g., Yeates 2007; Kreuzinger-Janik et al. 2021; Ahmed et al. 2024). On average, about 10 nematode species were found per TreM-type, and despite variability among TreMs, nematode species turnover across TreM-types was high, suggesting that, at the tree, and at the stand-level, a greater diversity of TreMs is associated to a higher nematode diversity. This finding supports forest management approaches that preserve large, old trees bearing diverse TreMs (aka. retention forestry; Torres-García et al. 2024), contributing to increased overall forest biodiversity.

### Nematode taxonomic and functional composition differs across TreMs

Nematodes dominated micro-invertebrate assemblages in most TreMs, yet community composition and functional traits varied between TreM-types. A particularly striking pattern was the difference between ‘external’ TreMs (e.g., mosses, lichens, ivy, epicormic shoots, burrs), which are exposed to fluctuating environmental conditions, and ‘internal’ TreMs (e.g., cavities, fungi), which are more buffered. Desiccation-resistant groups such as tardigrades, bdelloid rotifers, and plectid nematodes were more common in external TreMs, consistent with their known physiological adaptations to moisture, temperature, and UV stress (Rebecchi et al. 2020).

TreM physical structure also appeared to exert selective pressure on community composition; for instance, vermiform organisms like nematodes are well-adapted to forage in fine interstitial spaces (Erktan et al. 2020). Rather than large-scale biogeoclimatic gradients, it is often microgradients (e.g., local moisture and organic matter) that shape diversity patterns in TreMs (Paillet et al. 2017). We found evidence of bottom-up structuring of communities by substrate and micro-environmental properties. For example, internal TreMs typically supported simpler food webs dominated by bacterivorous and fungivorous nematodes, while external TreMs exhibited a higher proportion of omnivores and predators, and of organisms with more diverse body-shapes and sizes.

While our data did not challenge the hierarchical TreM classification of Larrieu et al. (2018), it does highlight additional axes of ecological differentiation within TreM-types taking the opportunity of the distinctive features offered by micro-invertebrate assemblages. For example: vertical structuring within substrates (e.g., layers within cavity detritus) could inspire finer TreM typologies resolved at centimetric scales. Likewise, using micro-invertebrates to document finer successional changes over time from formation of the TreM to its disappearance.

Crucially, sampling in this study was not replicated through time for each TreM-type. Different TreMs were collected during different seasons, so seasonal variation may be confounded with TreM-type differences. For instance, summer sampling (warmer and drier) versus autumn sampling (cooler with more organic inputs) could account for differences in moisture-sensitive taxa or trophic structure. Seasonality and resource turnover are known drivers of insect succession in rot-holes and fungal fruiting bodies (e.g., Stokland et al. 2012), and likely also affect nematode dynamics. These temporal dynamics, what we might call the"TreM tempo", should be addressed more cautiously in future studies with repeated sampling at shorter (e.g., weeks for ephemeral fungal fruiting bodies) or longer (e.g., seasons for cavities) intervals. Nevertheless, nematodes and other micro-invertebrates really offer practical advantages due to their ubiquity, fast life cycles, and minimal sampling requirements, making temporal replication at the individual TreM-scale feasible.

### Food webs and trophic pathways associated with TreMs

Isotopic niche analysis revealed clear trophic differentiation among TreM-forms, adding a valuable dimension to the TreM typology proposed by Larrieu et al. (2018). Micro-invertebrates occupied all trophic levels, in agreement with compelling evidences reported from other limno-terrestrial habitats (e.g., Schmid-Araya et al. 2016; Majdi and Traunspurger 2017; Wu et al. 2019; Hemmerling et al. 2022; Kreuzinger-Janik et al. 2022). This challenges the perception that tiny micro-invertebrates should just be confined to basal positions in food webs. Morphological traits such as stylets allow small invertebrates to feed across a wide range of prey sizes and types, complexifying trophic networks and reinforcing their importance as both consumers and predators.

Such complex trophic situations were especially apparent in bark-associated external TreMs (lichens, mosses, ivy, bark pockets), where it was possible to resolve isotopic differences among several nematode feeding groups. Stylet-bearing Dorylaimids occupied top-predator positions, as confirmed by enriched ^15^N signatures, and prior evidence of predation on other nematodes (e.g., Shafqat et al. 1987; Bilgrami 1995; Bilgrami et al. 2001). These Dorylaimids shared top-trophic positions with other stylet-bearing micro-invertebrates such as mites and tardigrades and with Mononchida nematodes which have large buccal cavities armed with sclerotized denticles. In contrast, bacterivorous Plectids occupied basal trophic positions, consistent with previous behavioral and isotopic studies (e.g., Hohberg and Traunspurger 2009; Kreuzinger-Janik et al. 2022).

In other TreMs such as fungal fruiting bodies or rot-holes, food webs were fueled directly by fungal tissues, or indirectly by plant detritus (wood, leaf litter), or insect droppings (aka.'frass'). Some Rhabditid nematodes in these habitats exhibited surprisingly high δ^15^N signatures despite not being known predators. We hypothesize that these nematodes may be opportunist scavengers or entomocoprophagous feeding on bacteria growing on insect droppings and remains, leading to disproportionate ^15^N enrichment via the microbial loop.

The case of woodpecker breeding cavities was particularly interesting. These cavities contained guano-rich substrates, leading to higher baseline δ^15^N signatures. They also harbored unusually high densities of *Mesorhabditis spiculigera*, which dominated nearly to the exclusion of other nematodes. This species, although notoriously bacterivorous, showed the highest δ^15^N signature of any other nematode in our study, likely due to bacterivory on guano-derived bacteria. Whether this reflects a specialized ornithocoprophagous lifestyle or just an opportunistic colonization of those woodpecker breeding cavities remains to be tested.

## Conclusion

This exploratory study highlights strong links between TreM typology and the species, traits, and trophic structure of micro-invertebrate communities, with a special focus on nematodes. We show that TreMs host rich assemblages of micro-invertebrates functioning as distinct"micro-ecosystems"with unique food web dynamics. These findings provide a valuable baseline; emphasizing the need for broader taxonomic surveys including temporal dynamics and occurences of TreMs across biomes, to better understand the role of TreMs and their associated communities in forest ecosystems worldwide. Such insights have the potential to better inform conservation strategies focused on old-growth forests and TreM-bearing trees as relevant biodiversity hotspots.

## Supplementary Information

Below is the link to the electronic supplementary material.

Supplementary file1 (DOCX 657 KB)

Supplementary file2 (XLSX 56 KB)
