# Supplementary material for: Tree-related microhabitats harbor distinct micro-invertebrate communities and support complex food webs
Source: Oecologia. 2025 Aug 21;207(9):148. doi: 10.1007/s00442-025-05774-5 (PMC12370837; doi:10.1007/s00442-025-05774-5)
Supplement: Supplementary file 1 — Supplementary file1 (DOCX 657 KB) [file 442_2025_5774_MOESM1_ESM.docx]

Supplementary material to:

**Tree-related micro-habitats harbor distinct micro-invertebrate communities and support complex food webs**

Nabil Majdi^1,2,3,4,*^, Walter Traunspurger^4^, Joseph Garrigue^1^ & Laurent Larrieu^5,6^

1 Massane Forest Reserve, Banyuls-sur-Mer, France.

2 Toulouse University, LRSV, UMR 5546 UPS/CNRS/INPT. Auzeville-Tolosane, France.

3 University of Applied Sciences and Arts of Southern Switzerland, Institute of Microbiology. Mendrisio, Switzerland.

4 Bielefeld University, Animal Ecology. Bielefeld, Germany.

5 Toulouse University, INRAE, UMR DYNAFOR, Auzeville-Tolosane, France.

6 CNPF-CRPF Occitanie, Tarbes, France.

* Corresponding author: nabil.majdi@univ-tlse3.fr / +33 787 48 19 55

**Table S1.** Types and forms of TreMs (after Larrieu et al. 2018) collected in Massane forest natural reserve. Abbreviations: N, number of samples collected from different trees. Morphology, morpho-taxonomy of micro-invertebrates and nematodes. SIA, Stable isotopic analysis.

| TreM-forms | Abbrev. | TreM-types | Abbrev. | Season | Sampling procedure | N | Analyses |
| --- | --- | --- | --- | --- | --- | --- | --- |
| Fruiting bodies of saproxylic fungi and slime moulds | Fungi | Pulpy agaric (*Hericium clathroides*) | *Hericium* | Autumn | Hand-detached or slightly scraped with a knife | 3 | Morphology, SIA |
|  |  | Annual polypore (*Inonotus cuticularis*) | *Inonotus* | Autumn | Hand-detached or slightly scraped with a knife | 3 | Morphology, SIA |
|  |  | Perennial polypore (*Fomes fomentarius*) | *Fomes* | Spring | Hand-detached or slightly scraped with a knife | 3 | Morphology, SIA |
|  |  | Annual polypore (*Trametes* sp.) | *Trametes* | Autumn | Hand-detached or slightly scraped with a knife | 3 | Morphology, SIA |
|  |  | Large pyrenomycete | Pyrenomycete | Autumn | Scraping 10 cm^2^ patches of bark with a knife | 3 | Morphology |
| Cavities | Cavities | Insect galleries and bore holes | Insect-Gallery | Spring | Digging (2 cm depth) with shovel and spoon | 3 | Morphology |
|  |  | Large woodpecker breeding cavity* | WPNest | Summer | Climbing (5-8 m), digging (2 cm depth) with spoon | 3 | Morphology, SIA |
|  |  | Trunk rot-hole | Rot-hole | Spring | Digging (2 cm depth) with shovel and spoon | 3 | Morphology, SIA |
| Tree injuries and exposed wood | Wounds | Bark pocket | Bark-pocket | Spring | Scraped with a spoon (<1 cm depth) | 3 | Morphology, SIA |
|  |  | Limb breakage | Limb-breakage | Summer | Detachment with saw, crumbling with axe | 3 | Morphology |
| Epiphytic and parasitic crypto- and phanerogames | Epiphytic | Bryophytes | Moss | Spring | Hand-detached or slightly scraped with a knife | 3 | Morphology, SIA |
|  |  | Crown microsoil | Crown-MS | Summer | Scraped with a spoon (<1 cm depth) | 3 | Morphology, SIA |
|  |  | Foliose and fructicose lichens | Lichen | Spring | Hand-detached or slightly scraped with a knife | 3 | Morphology, SIA |
|  |  | Bird nest** | Nest | Summer | Hand-detached | 3 | Morphology |
|  |  | Ivy and lianas | Ivy | Summer | Hand-detached or slightly scraped with a knife | 3 | Morphology, SIA |
| Excrescences | Excrescences | Burr | Burr | Summer | Scraped with knife and axe | 3 | Morphology |
|  |  | Epicormic shoots | Epicormic-shoots | Autumn | Trapped litter scraped with a spoon | 3 | Morphology |
| Crown deadwood | Deadwood | Dead branches | Dead-branches | Autumn | Detachment with saw, crumbling with axe | 3 | Morphology |
| * unoccupied >1 yr, 1 *Dryocopus martius* cavity, 2 *Picus sharpei* cavities | | |  |  |  |  |  |
| ** unoccupied >1 yr, nests of 3 different species: *Turdus viscivorus, Troglodytes troglodytes* and *Erythacus rubecola* | | | | | |  |  |

**Micro-invertebrate extraction procedure**

After field collection, TreM samples were brought to the laboratory and placed in fine gauze cloth (approx. 500 μm mesh), which was laid on a steel grid positioned in the upper part of a large funnel (diameter 40 cm; modified Baermann funnel). The funnels were filled with water so that the water surface slightly covered the steel grid, allowing the TreM samples in the gauze to rest at the air–water interface. Hydrophilic microfauna (e.g., nematodes, rotifers, and tardigrades) were expected to rehydrate from anhydrobiosis and actively migrate through the gauze and grid into the water phase, eventually sinking to the bottom of the funnel. After 48 hours of extraction, a duration sufficient to allow active migration while minimizing in-funnel reproduction or predation (Travé et al. 1954), the entire water phase was sieved through a 20 μm mesh. This 'live' extraction method (so-called because most organisms remained alive at the time of fixation) proved highly effective for isolating nematodes, tardigrades, rotifers, mites, and small insect larvae. It yielded relatively clean samples, thereby simplifying sorting, counting, and cleaning procedures. Additionally, microscopic examination of gut contents revealed that most extracted individuals had starved during the extraction period, which improves the reliability of stable isotope analysis by minimizing bias from undigested food residues in their guts.

**Stable Isotope Analyses**

Fine and coarse particulate organic matter (FPOM and CPOM), small fragments of leaf litter, and wood particles were manually sorted under a stereomicroscope from each bulk TreM sample. These materials were then homogenized into a fine powder using an Ultra Turrax T-8 disperser (IKA, Staufen, Germany) to represent the baseline organic resources available within the TreM. Concurrently, macroinvertebrates were sorted, identified to the lowest feasible taxonomic level, and large individuals were similarly homogenized and encapsulated for stable isotope analysis.

Frozen biological samples were thawed prior to processing. Individual or grouped specimens were carefully handled using an eyebrow hair mounted on a needle or a flame-thinned Pasteur pipette connected to a mouthpiece. To ensure removal of external contaminants, organisms were rinsed twice by successive transfers through Petri dishes containing 10 mL of Milli-Q water. After cleaning, specimens were grouped (10–50 individuals, depending on size) and transferred in a ~10 μL volume, which was placed into a pre-weighed tin capsule for drying and analysis. Given the minimum tissue mass required for reliable SIA (~0.1 mg dry weight), multiple individuals were often pooled (e.g. 200–300 nematodes, 10–50 dipteran larvae) to meet detection thresholds of the elemental analyzer.

Elemental carbon (C) and nitrogen (N) content and stable isotopic ratios (δ¹³C and δ^15^N) were determined using a NA1110 elemental analyzer coupled to a DELTA Plus mass spectrometer via a Conflo III interface (Finnigan MAT, Bremen, Germany). Organic C and N percentages were calculated relative to total dry organic matter. Isotopic values were expressed in ‰ using the delta notation: δX (‰) = [(R_sample / R_standard) – 1] × 1000,

where X is either ¹³C or ^15^N, and R represents the corresponding heavy/light isotope ratio. Vienna Pee Dee Belemnite (VPDB) and atmospheric N_2_ were used as the international standards for C and N, respectively. Acetanilide (Thermo) and peptone (Sigma-Aldrich) were used as internal calibration standards throughout the analytical runs. Measurement precision was better than ±0.15‰ for both δ¹³C and δ¹⁵N, in accordance with Thermo specifications.

**Interpretation of δ¹³C and δ^15^N Signatures in Ecological Context**

Stable isotope analysis of carbon (δ¹³C) and nitrogen (δ^15^N) provides insight into organisms’ trophic positioning and dietary sources. δ¹³C values generally reflect the origin of primary production, distinguishing between sources such as microalgae versus detrital or microbial food webs. In contrast, δ^15^N values typically increase with trophic level due to predictable isotopic enrichment during metabolic assimilation. Here, we applied standard trophic enrichment factors of +0.4‰ (±1.3‰ SD) for δ¹³C and +3.4‰ (±1.0‰ SD) for δ^15^N between consumers and their assimilated food sources (Post 2002). These values are widely used in ecological studies, despite some variation depending on taxa and metabolic pathways (e.g., McCutchan et al. 2003). Using this framework, we identified potential predators as those with elevated δ^15^N values compared to other community members (Fig. S1). δ¹³C values, in turn, allowed us to infer the dominant basal energy pathways (e.g., whether organic matter was derived from primary producers or decomposed terrestrial inputs).

**Stable Isotope Signatures of Sources and Consumers**

The bulk isotopic signatures of invertebrates and food sources were positioned in a C-N biplot, highlighting different trophic positions between the different TreM food webs (Fig. S1).

Within the 'cavities' TreM-form (Fig. S1A), the TreM-types rot-hole and woodpecker breeding-cavity showed distinct trophic positions, based on ^15^N-enriched signature of the basal organic matter in the woodpecker breeding cavities. Dipteran larvae (Chironomidae, Mycetophilidae, Scatopsidae) thrived on this resource. Sharing a dominant position in this food web were predacious dipteran larvae (Ceratopogonidae) and the bacterivorous nematode species *Mesorhabditis spiculigera*. However, Syrphidae larvae showed ^13^C-enriched isotopic signature in comparison to other invertebrates of woodpecker breeding cavities, suggesting contrasted resource use. In rot-holes, the entire invertebrate community showed isotopic signatures consistent with that of the basal CPOM. Bacterivorous rhabditid nematodes showed enriched ^15^N signature and thus a top position in the biplot.

In bark pockets (Fig. S1B), the signature of leaf litter resource was ^13^C-depleted in comparison to other CPOM, Adult beetles and Dorylaimid nematodes showed enriched ^13^C signatures in comparison to other invertebrates.

In Mosses and lichens, the signature of basal resources differed: Lichens' CPOM showing substantially ^15^N-depleted and ^13^C-enriched values in comparison to mosses' CPOM (Fig. S1C). There, predacious Mononchidae nematodes and mites occupied top positions, tardigrades and Dorylaimidae nematodes occupied intermediary positions, and Plectidae nematodes were basal consumers showing most depleted ^15^N signatures.

Similar isotopic niches could be observed for ivy's aerial roots (Fig. S1D), but in crown microsoils, the fine-particulate organic matter (FPOM), Chironomidae and Empididae larvae showed depleted ^13^C signatures.

Within the TreM-form ‘fungal fruiting bodies’, the TreM-type perennial polypore represented by *Fomes fomentarius* (Fig. S1E), showed a relatively simple architecture with Fomes' CPOM as a basal resource, then nematodes, then adults of *Bolitophagus* spp. occupying top-position in the food web. In contrast, *Inonotus* (annual polypores) and their associated food webs showed more enriched ^15^N-signatures, with small Rhabditidae nematodes occupying top-position (Fig. S1E).

Food web structure was comparable in other fungi, but resource and consumer signatures were more ^15^N-depleted and ^13^C-enriched in *Hericium* in comparison to *Trametes* (Fig. S1F).

**Figure S1.** Mean and standard deviation (N =3) of bulk isotopic signatures of resources and invertebrates in a different types of Tree-related Microhabitats (TreMs) grouped by forms (A): Cavities, (B): Wounds, (C–D): Epixylic and epiphytic structures, (E–F): Fruiting bodies of saproxylic fungi. Abbreviations: CPOM: Coarse Particulate Organic Matter of the TreM habitat (habitat as resource). FPOM: Fine Particulate Organic Matter of the TreM (organic particles passing through a 1 mm sieve). Frass: Recognizable insect excrements.

**Isotopic Niche Metrics and the SIBER Framework**

To quantify isotopic niche breadths and overlaps, we calculated community-wide metrics originally proposed by Layman et al. (2007), including:

CR: δ¹³C range (proxy for basal resource diversity)

NR: δ^15^N range (proxy for trophic diversity)

TA: Total area of the convex hull in δ¹³C–δ^15^N space

CD: Mean Euclidean distance of each group to the centroid (indicative of trophic diversity)

NND: Mean nearest-neighbor distance (indicator of trophic redundancy)

SDNND: Standard deviation of NND (indicator of trophic evenness)

These metrics were further analyzed using the Stable Isotope Bayesian Ellipses in R (SIBER package), which reformulates Layman's metrics within a Bayesian framework. Unlike traditional bootstrapping, the Bayesian approach incorporates uncertainty and error in sampling, providing posterior distributions rather than point estimates or p-values.

Posterior means were estimated via a Markov Chain Monte Carlo (MCMC) approach using JAGS (Plummer 2003), with 2 chains run for 200,000 iterations, a burn-in of 10,000 iterations, and thinning by a factor of 10. The resulting posterior distributions allowed us to derive multivariate ellipse-based metrics such as SEA.B (Bayesian standard ellipse area) and reformulated Layman.B indices, enabling robust comparisons of isotopic niche space among communities.

We used these metrics to compare isotopic niches across both resource types (CPOM, microfauna, macrofauna) and TreM types ("cavities", "fungi", and "epiphytes"), providing a probabilistic evaluation of niche differentiation and overlap among and within microhabitat communities.

**Table S2** Average (N= 3) relative abundance, diversity and functional indices of nematode species in 18 different types of tree-related microhabitats (TreMs) associated with European beech in the Massane forest reserve (eastern French Pyrenees). Significant results of a multi-level pattern analysis (MULTIPATT) associating a given species with one or more forms or types of TreMs are shown. An electronic .xls version of this table is provided as supplement.

**Literature cited**

Larrieu L, Paillet Y, Winter S, et al (2018) Tree related microhabitats in temperate and Mediterranean European forests: A hierarchical typology for inventory standardization. Ecological Indicators 84:194–207

Layman CA, Arrington DA, Montaña CG, Post DM (2007) Can Stable Isotope Ratios Provide for Community-Wide Measures of Trophic Structure? Ecology 88:42–48

McCutchan JH, Lewis WM, Kendall C, McGrath CC (2003) Variation in trophic shift for stable isotope ratios of carbon, nitrogen, and sulfur. Oikos 102:378–390

Plummer M (2003) JAGS: A program for analysis of Bayesian graphical models using Gibbs sampling. In: Proceedings of the 3rd international workshop on distributed statistical computing. Vienna, Austria, pp 1–10

Post DM (2002) Using stable isotopes to estimate trophic position: models, methods, and assumptions. Ecology 83:703–718

Travé J, Gadea E, Deboutteville C (1954) Contribution à l'étude de la faune de la Massane (Première Note). Vie et Milieu 5:201–214
